# Measurement Properties of the Attitudes to Gene Therapy for the Eye (AGT-Eye) Instrument for People With Inherited Retinal Diseases

**DOI:** 10.1167/tvst.11.2.14

**Published:** 2022-02-08

**Authors:** Myra B. McGuinness, Alexis Ceecee Britten-Jones, Lauren N. Ayton, Robert P. Finger, Fred K. Chen, John Grigg, Heather G. Mack

**Affiliations:** 1 Centre for Eye Research Australia, Royal Victorian Eye and Ear Hospital, Melbourne, Victoria, Australia; 2 Centre for Epidemiology and Biostatistics, Melbourne School of Population and Global Health, University of Melbourne, Melbourne, Victoria, Australia; 3 Department of Optometry and Vision Sciences, University of Melbourne, Melbourne, Victoria, Australia; 4 Ophthalmology, Department of Surgery, University of Melbourne, Melbourne, Victoria, Australia; 5 Department of Ophthalmology, University of Bonn, Bonn, Germany; 6 Centre for Ophthalmology and Visual Sciences (incorporating Lions Eye Institute), The University of Western Australia, Perth, Western Australia, Australia; 7 Royal Perth Hospital and Perth Children's Hospital, Perth, Western Australia, Australia; 8 Save Sight Institute, The University of Sydney, Sydney, New South Wales, Australia; 9 Eye Genetics Research Unit, Sydney Children's Hospitals Network, Save Sight Institute, Children's Medical Research Institute, University of Sydney, Sydney, New South Wales, Australia

**Keywords:** ocular gene therapy, inherited retinal diseases, questionnaire

## Abstract

**Purpose:**

To assess the measurement properties of the Attitudes to Gene Therapy for the Eye (AGT-Eye) instrument among Australian adults with inherited retinal diseases (IRDs) and parents/caregivers of people with IRDs. Constructs of interest included sources of information, knowledge of treatment methods, awareness of treatment outcomes, and perceived value of gene therapy for IRDs.

**Methods:**

A cross-sectional, self-reported, 30-item questionnaire was administered in English from January to June 2021. It was predominantly conducted online with phone and paper alternatives available. Rating scale models were generated separately for each of the four subscales to assess fit, discrimination, and differential item functioning of the items, as well as targeting, reliability, and precision of the subscales. Principal components analysis was used to assess dimensionality.

**Results:**

Responses from 681 participants (87.1% online, 12.9% phone/mail) were included (ages 18–93 years; 51.7% female). Removal of two poorly performing items slightly improved subscale properties. Item reliability was high for each of the subscales; however, person reliability was suboptimal, with limited ability to stratify participants according to traits (person separation coefficient < 1.8 for each subscale). There was no evidence of differential item functioning by gender, online completion, or patient/caregiver status. Evidence of multidimensionality was detected for two subscales.

**Conclusions:**

Four subscales of the AGT-Eye will be used to analyze operational knowledge and perceived value of ocular gene therapy in Australia. Measurement properties may be improved with the generation of additional items.

**Translational Relevance:**

Physicians can use the AGT-Eye to assess knowledge and expectations of potential recipients of ocular gene therapy for IRDs.

## Introduction

Following the approval of any novel health intervention for clinical use, patient awareness of the possible risks and benefits is likely to lag behind that of established treatments. As the availability of approved gene therapies for inherited retinal diseases (IRDs) is projected to increase over the next decade, so are the information needs of prospective recipients.^1^ Treating physicians have a responsibility to inform patients of potential benefits and risks and to correct misunderstandings prior to any procedure. However, until now, there have been no validated instruments designed to ascertain the level of knowledge about ocular gene therapy among people with IRDs.

Retinal disorders caused by inherited gene mutations are heterogeneous, both genetically and phenotypically.^2^^–^^4^ Although monogenic IRDs are considered rare orphan conditions, combined they represent one of the most common causes of legal blindness in adults of working age in developed nations.^5^^–^^7^ IRDs have a significant impact on quality of life and a substantial economic cost for affected individuals and the healthcare system.^8^^–^^10^ Hence, therapies that seek to assuage the burden of IRDs on individuals, families, and the healthcare system are in demand.

Instruments are available to assess patient-reported outcomes and perceptions among participants enrolled in clinical trials.^11^^–^^14^ Indeed, perspectives and decision-making processes have been investigated among participants in trials of gene therapy for *RPE65* retinopathy, choroideremia, and X-linked retinoschisis.^15^^–^^18^ However, there is limited information on consumer expectations for gene therapy interventions that have received approval for clinical use from a regulatory body, and these expectations are hypothesized to be higher than those for experimental interventions.^19^

The Attitudes to Gene Therapy for the Eye (AGT-Eye) is an English-language, 30-item knowledge, attitude, and practice (KAP)-like survey specifically designed to investigate the knowledge and perceived value of ocular gene therapy interventions that have been approved for patients with IRDs, or will be approved in the future.^20^ The AGT-Eye attempts to capture the latent traits of knowledge about methods, awareness of potential treatment outcomes, and perceived value of gene therapy for IRDs. In addition, it aims to capture the current sources of information about gene therapy. The aim of this paper is to investigate the measurement properties of the AGT-Eye among adults with IRDs, the parents and guardians of minors with IRDs, and caregivers of adult dependents with IRDs in Australia.

## Methods

The AGT-Eye was administered as part of a cross-sectional survey conducted with non-random sampling.^20^ The self-reported survey was administered in English, predominantly as an online questionnaire. Paper-based and phone interview alternatives were available on participant request, and responses were transcribed by study staff. Deidentified data were securely captured and managed using the REDCap electronic data capture tool, hosted by the Centre for Eye Research Australia.^21^ This study was conducted in compliance with the tenets of the Declaration of Helsinki and was approved by the Human Research Ethics Committee of the University of Melbourne (2057534). After providing information on the nature of the study, consent was obtained from each participant.

### Participants

For this study, IRDs (including syndromic forms) were defined as retinal disorders caused by an inherited/spontaneous gene mutation resulting in loss of photoreceptor function accompanied by visual loss. Australians at least 18 years of age with an ophthalmologist-diagnosed IRD (as self-reported) were eligible, as were adult guardians of minors (<18 years old) with an IRD and caregivers of adult patients who did not have the capacity to undertake the survey. As the person responsible for making treatment decisions, parents, guardians, and caregivers were asked to respond according to their own perceptions (rather than those of their children or dependents). IRD mutation carriers without ocular signs or symptoms of IRDs were excluded. People with other retinal conditions with known genetic risk factors (such as age-related macular degeneration) were excluded in the absence of an IRD. Recruitment material (emails, postal mail, or social media) were distributed by the Australian Inherited Retinal Disease Registry and DNA Bank,^22^ the Centre for Eye Research Australia, Royal Australian and New Zealand College of Ophthalmologists, patient support groups (Retina Australia, Vision Australia, Cure Blindness Australia and UsherKids Australia), ophthalmology and clinical genetics departments of metropolitan tertiary hospitals, and private practices of IRD subspecialists. The survey was open for completion between January and June 2021, without a cap on respondent numbers.

### Demographics

Demographic information, including age and gender of respondent, type of IRD (checkbox with free text option for other), education level, and household income, and self-reported clinical data were captured prior to administering the AGT-Eye. Following completion of the AGT-Eye, three other structured instruments were administered: EuroQol EQ-5D-5L,^23^ National Eye Institute Visual Function Questionnaire (NEI VFQ-25),^24^ and Patient Attitudes to Clinical Trials (PACT22).^11^ Responses to these instruments and their correlation with AGT-Eye items will be reported in a separate publication.

### AGT-Eye Questionnaire

As previously described, themes and domains were initially generated by a panel of expert IRD subspecialist ophthalmologists and clinical geneticists.^20^ Item refinement occurred following consultation with patient focus groups and content matter and research experts.^20^ Each item had five response options ranging from 1 (*Strongly disagree*) to 5 (*Strongly agree*). The AGT-Eye was designed as a multidimensional KAP-like survey with items as listed in Supplementary Table S1.^25^ Sources of information about gene therapy were captured in items 2a to 2i (Subscale A). Items 1 and 3 to 7 (Subscale B) contain statements about self-perceived knowledge and the timing and method of treatment. Currently, item 5 is considered to be a true statement, and items 4, 6, and 7 are considered false. Items 8 to 17 (Subscale C) contain statements related to awareness of potential outcomes that cannot be fully predicted. However, items 8, 12, and 16 are considered more likely to be false than true. People who agree with the statements in items 8 and 9 are considered to have positive expectations of outcomes, whereas agreement with the remaining items in this subscale indicate negative expectations. The final subscale (D, items 18–22) relates to the perceived value of gene therapy treatment.

### Assessment of Measurement Properties and Recalibration

The natural grouping of items was first investigated through factor analysis prior to fitting item response theory models (responses coded 1–5). For this, the principal-factor method was used to analyze the correlation matrix (i.e., the pairwise correlation between responses from each item). Factors (combinations of different item loadings) with a strength of ≥1 eigenvalue were considered the most informative item groupings. Items with loadings of ≤−0.3 or ≥0.3 were considered influential on a factor (i.e., responses to the item correlated with responses to other influential items in that factor). Items with uniqueness (residual value from the factor model) > 0.6 across retained factors were not considered well explained by these factors (i.e., items did not fit well into any of the identified groups of items).

Scores from items 4, 6 to 8, 12, and 16 were then reverse coded so that higher scores indicated a greater level of knowledge, and item response models were fit separately for each of the four subscales. Rating scale models were fit for Subscales A and D, and grouped rating scales were fit for Subscales B and C (items grouped according to order of coding). As recommended for KAP surveys (and in recognition that the perceived value of treatment could plausibly increase or decrease with higher levels of information and knowledge), subscale scores were not combined to generate a total AGT-Eye score.^25^^,^^26^

Item infit mean-square (i.e., the fit of the item response model to observed participant responses) was assessed using weighted standardized model residuals. Items with infit of <0.7 were considered overfitted (highly correlated to other items), and items with infit of >1.3 were considered underfitted (considerable variation). Item discrimination was considered low (i.e., discriminates between high and low performers less than expected for an item of this measure) if it was <0.6. The ability of the subscales to stratify participants (scale precision) was quantified using the person separation coefficient (PSC). A PSC of 1.25 indicates that participants can be stratified into two groups, whereas a PSC of 2.00 indicates that participants can be stratified into low, medium, and high levels of the trait. Person reliability, which also provides a measure of discrimination, was estimated using Cronbach's α (≈ 2–3 levels of people if α = 0.8, ≈1–2 levels if α = 0.5). The average person measure for each subscale was used to assess the level of targeting of the subscale to this cohort, with <1 logit considered appropriate. Item reliability was derived from the observed variance of item difficulties and the mean of squared standard errors of item difficulty measures. Differential item functioning (DIF) was assessed according to gender (male vs. female), survey type (online vs. paper/phone), and respondent status (adult vs. parent, guardian, or caregiver). DIF contrasts ≥ 1 logit were considered evidence of differential functioning. Principal components analysis of model residuals was used to investigate the variation in responses and multidimensionality (i.e., eigenvalue of unexplained variance in first contrast ≥ 2); contrast loadings < −0.4 or > 0.4 were considered large. Poorly performing items (as defined above) were removed to explore potential improvements in instrument properties. As an indicator of concurrent validity, Spearman's correlation coefficient was used to assess correlation between recalibrated subscale scores and between subscale scores and selected demographic and instrument items.

### Statistical Methods

Participants who completed the AGT-Eye online in ≤30 seconds were excluded, as this speed was thought to be incompatible with genuine consideration of each item. Complete-case analyses were conducted (i.e., only participants with non-missing data on all variables were included). Type of IRD was classified by an IRD subspecialist ophthalmologist (HGM) as being associated with photoreceptor loss predominantly in the macular or widespread across the retina (as listed in Supplementary Table S2). Demographics were compared between included and excluded respondents and between included respondents who completed surveys online and those with phone or paper surveys using two-sample *t*-tests (for age) and Pearson's χ^2^ tests (for categorical variables). Descriptive statistics, factor analysis, and graphs were produced using Stata/BE 17.1 (StataCorp, College Station, TX). Measurement properties were assessed using Winsteps 4.6.2 (Beaverton, OR).

## Results

### Participants

Consent was obtained for 1036 surveys (*n* = 932 online, *n* = 8 via telephone, *n* = 96 paper), and demographic data was provided on 857 surveys. From these, 139 surveys (16.2%) were incomplete, two respondents (0.2%) were <18 years of age, 26 respondents (1.9%) had no eligible IRD or the broad type of IRD was unknown, and three participants (0.4%) completed the AGT-Eye in ≤30 seconds (see Supplementary Table S3 for a comparison of the demographics between included and excluded surveys). Responses from 681 participants (79.5%) were included in the analyses (*n* = 593 [87.1%] online, *n* = 8 [1.2%] via telephone, *n* = 80 [11.7%] paper). Of the included respondents, 639 (93.8%) reported having an IRD themselves, and 42 (6.2%) were the parent, guardian, or caregiver of a person with an IRD (see Table 1). Respondents were 18 to 93 years of age (mean ± SD, 53.5 ± 15.8 years), and just over half of the respondents were female (*n* = 352; 51.7%). Time to complete the AGT-Eye portion of the online survey ranged from 34 seconds to 4 minutes (median, 1 minute, 57 seconds).

**Table 1.** AGT-Eye Respondent Characteristics

	Phone or Paper	Online	Total	
	(*n* = 88)	(*n* = 593)	(*n* = 681)	*P* ^a^
Age of respondent (y)				<0.001
Range	25–91	18–93	18–93	
Mean (SD)	64.6 (14.4)	51.8 (15.4)	53.5 (15.8)	
Respondent status, *n* (%)				0.249
Adult patient	85 (96.6)	554 (93.4)	639 (93.8)	
Parent, guardian, or caregiver	3 (3.4)	39 (6.6)	42 (6.2)	
Gender, *n* (%)				0.720
Male	45 (51.1)	282 (47.6)	327 (48.0)	
Female	43 (48.9)	309 (52.1)	352 (51.7)	
Non-binary	0 (0.0)	2 (0.3)	2 (0.3)	
Vision loss status, *n* (%)				0.094
Widespread	74 (84.1)	451 (76.1)	525 (77.1)	
Macular	14 (15.9)	142 (23.9)	156 (22.9)	
Highest level of education completed, *n* (%)				<0.001
Primary school	6 (6.8)	9 (1.5)	15 (2.2)	
Secondary school (year 10 or above)	41 (46.6)	182 (30.7)	223 (32.7)	
Trade certificate	23 (26.1)	107 (18.0)	130 (19.1)	
Bachelor's degree	10 (11.4)	162 (27.3)	172 (25.3)	
Post-graduate degree	4 (4.5)	122 (20.6)	126 (18.5)	
I prefer not to say	4 (4.5)	11 (1.9)	15 (2.2)	
Gross annual household income, *n* (%)				<0.001
Less than $18,200	7 (8.0)	35 (5.9)	42 (6.2)	
$18,201–$37,000	32 (36.4)	84 (14.2)	116 (17.0)	
$37,001–$87,000	20 (22.7)	148 (25.0)	168 (24.7)	
$87,001–$180,000	9 (10.2)	176 (29.7)	185 (27.2)	
More than $180,001	1 (1.1)	60 (10.1)	61 (9.0)	
I prefer not to say	19 (21.6)	90 (15.2)	109 (16.0)	
Would receive gene therapy if available now, *n* (%)				0.018
Very unlikely	1 (1.1)	8 (1.3)	9 (1.3)	
Unlikely	2 (2.3)	3 (0.5)	5 (0.7)	
Neutral	3 (3.4)	40 (6.7)	43 (6.3)	
Likely	25 (28.4)	97 (16.4)	122 (17.9)	
Very likely	57 (64.8)	445 (75.0)	502 (73.7)	

a Two-sample *t*-test (age) and Pearson's χ^2^ test (categorical variables).

### Item Responses

**Figure 1.**
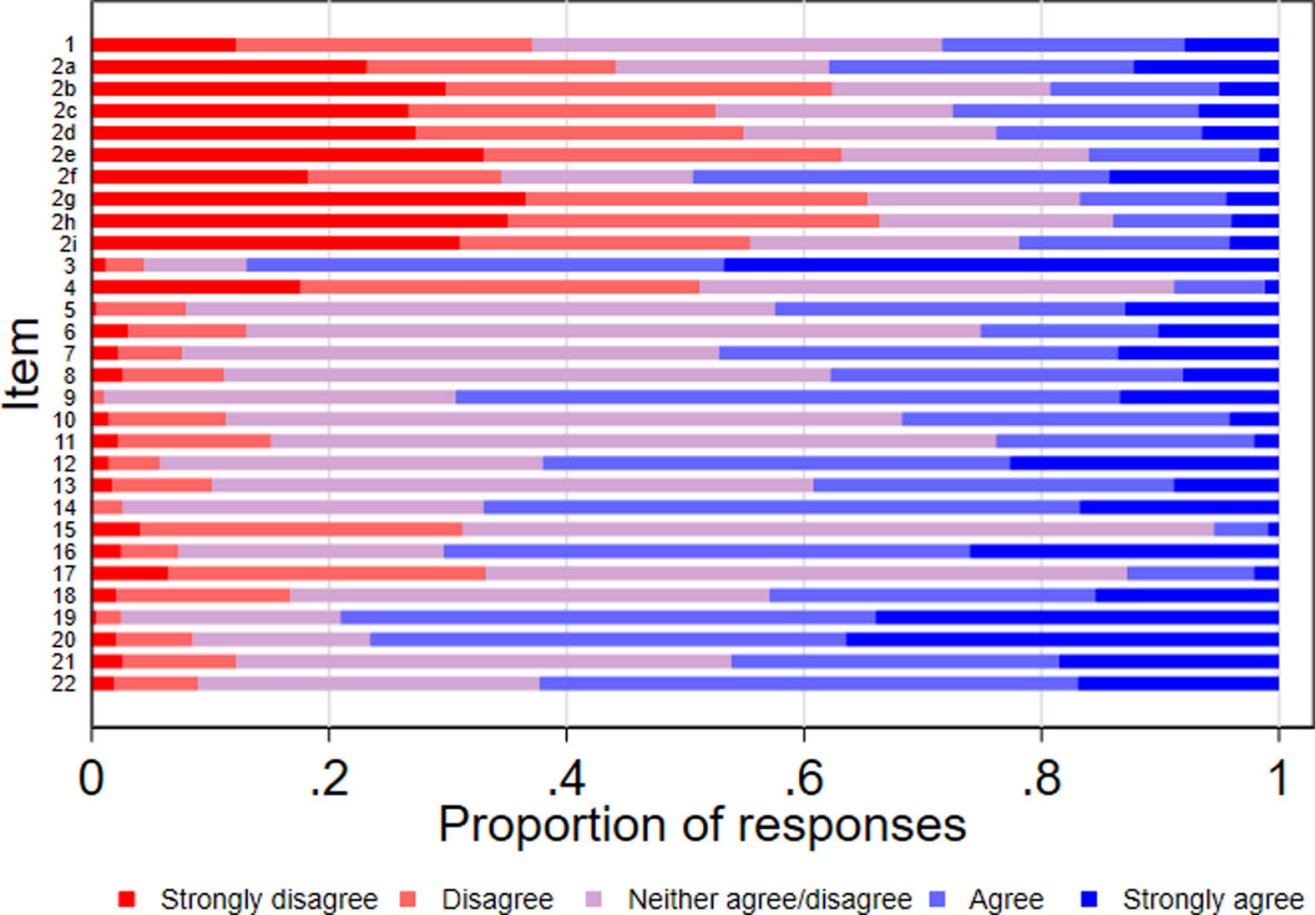
AGT-Eye item response frequencies. Scores from items 4, 6 to 8, 12, and 16 were reverse coded prior to plotting.

No floor effect was observed for any of the subscales; however, 46.7% strongly agreed with item 3 (*I understand the difference between an experimental treatment provided in a clinical trial and a treatment that has already been approved by the Australian Government*), suggesting a ceiling effect (Fig. 1, Supplementary Table S4). The median response category in Subscale A (*Sources of information*) was *Disagree*, indicating that respondents were not obtaining information on gene therapy from multiple sources. The median response for Subscales B and C was *Neither agree nor disagree* (Table 2), and over 50% of participants responded *Neither agree nor disagree* for 7/21 of the items in Subscales B to D.

**Table 2.** Item Properties of the AGT-Eye Instrument (*n* = 681 Respondents)

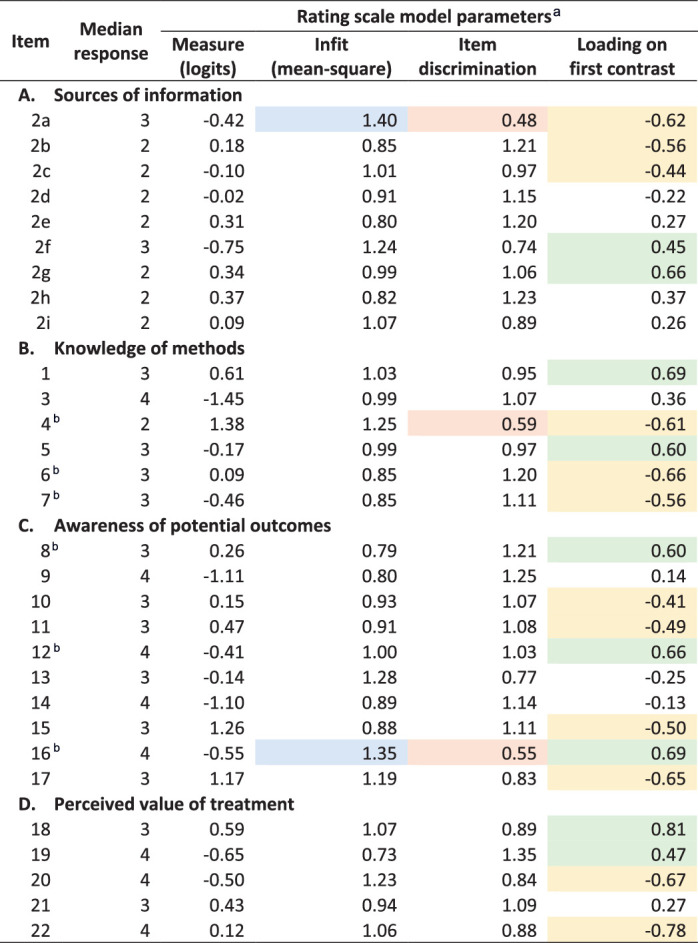

Shading indicates values outside preferred range: infit < 0.7 or > 1.3 (blue), discrimination < 0.6 (orange), loading < −0.4 (yellow) or > 0.4 (green).

a Separate item response theory model fitted for each subscale: rating scale models for Subscale A and E; grouped rating scale models for Subscales B and C.

b Responses reverse coded prior to analysis.

Four factors had an eigenvalue greater than 1 and explained 93.8% of the total variance between responses on factor analysis of the responses prior to fitting item response theory models (Supplementary Table S5). Unrotated loadings on the first factor were highest for item 1 (*I have good knowledge about gene therapy for inherited retinal diseases*) and the items in Subscale A (*Sources of information*) (Supplementary Table S6). Factor 3 was positively associated with items in Subscale D (*Perceived value of treatment*) and negatively associated with items related to Subscale A. Items in Subscale B (*Knowledge of methods*) and Subscale C (*Awareness of outcomes*) had higher loadings across factors 2 and 4. Uniqueness was >0.6 for all items except items 1 and 2b to 2i, indicating that most items were not well explained by these four factors.

### Measurement Properties

Subscales A to C appropriately targeted the level of participant knowledge (mean person scores, −0.78 to 0.52 logits), whereas participants showed higher levels of agreement with statements in Subscale D (*Perceived value of treatment*; mean person score, 1.14 logits) (see Table 3 and person-item maps in Supplementary Fig. S1). Only Subscale A (*Sources of information*) could be used to stratify participants into high and low levels of the trait of interest (PSC = 1.78; 0.57–1.04 for remaining subscales), and further evidence of suboptimal discrimination was provided by low levels of person reliability (α = 0.76 for Subscale A; α = 0.24–0.52 for Subscales B–D). Item reliability was extremely high for each of the subscales (≥0.98), indicating that the items had a sufficient range of difficulty and that the sample size was large enough to verify the item hierarchy (i.e., saturation was reached). Disordered Andrich response thresholds were observed for the reverse coded items within Subscale C (*Awareness of outcomes*) (see category response curves in Supplementary Fig. S2). There was no evidence of DIF by gender (contrast, −0.28 to 0.35), online completion (−0.47 to 0.56), or respondent as patient versus parent, guardian, or caregiver status (−0.43 to 0.74) within any of the subscales. Evidence of multidimensionality was present for Subscales B to D (eigenvalues of 2.1, 2.4, and 2.0, respectively) (see loading plot in Supplementary Fig. S3). Each model explained a moderate proportion of raw variance in the responses (33.1%–44.7%).

**Table 3.** AGT-Eye Subscale Properties (*n* = 681 Respondents)

	Subscale^a^			
	A	B	C	D
	Sources of	Knowledge of	Awareness of	Perceived
	Information	Methods	Outcomes	Value
**Original items**				
Number of items, *n*	9	6	10	5
Person discrimination				
Root mean square error	0.67	0.59	0.50	0.75
Separation coefficient	1.78	0.62	0.57	1.04
Strata of person abilities	2.7	1.2	1.1	1.7
Reliability				
Person (α)	0.76	0.28	0.24	0.52
Item	0.98	1.00	1.00	0.99
Targeting, mean person score (logits)	−0.79	0.39	0.52	1.14
Principal components analysis				
Raw variance explained (%)	41.9	44.7	33.1	37.5
Eigenvalue of first contrast	1.9	2.1	2.4	2.0
**Recalibration**				
Number of items	Unchanged	5	9	Unchanged
Person discrimination				
Root mean square error		0.71	0.55	
Separation coefficient		0.87	0.78	
Strata of person abilities		1.5	1.4	
Reliability				
Person (α)		0.43	0.33	
Item		1.00	1.00	
Targeting, mean person score (logits)		0.71	0.50	
Principal components analysis				
Raw variance explained (%)		44.7	35.1	
Eigenvalue of first contrast		1.9	2.1	

a Parameters estimated via a separate item response theory model for each subscale: rating scale models for Subscale A, B, and E; grouped rating scale models for Subscales C and D.

### Recalibration

Item 2a (*I have obtained information about gene therapy from my ophthalmologist*) had high infit and low discrimination compared with other items in Subscale A (*Sources of information*) (Table 2). However, this item was not removed because ophthalmologists will play a key educational role for people who do eventually present to discuss gene therapy. Higher infit and lower discrimination were also observed for item 4 (*Gene therapy for the eye is suitable at any stage of a person's life*) and item 16 (*I will lose my privacy if I undergo gene therapy, and my data will be in the public domain*). After removing these items from Subscales B and C, respectively, there was less evidence of multidimensionality, and PSC and person reliability improved slightly (Table 3). None of the remaining items in these subscales showed evidence of over- or underfitting. However, disordered Andrich response thresholds were observed for the reverse-coded items within both subscales (Supplementary Table S4), indicating some dysfunction exists for the groups of items consisting of “false” statements. This was not able to be resolved without removing the reverse-coded items.

### Correlation Between Subscale Scores and Items

**Figure 2.**
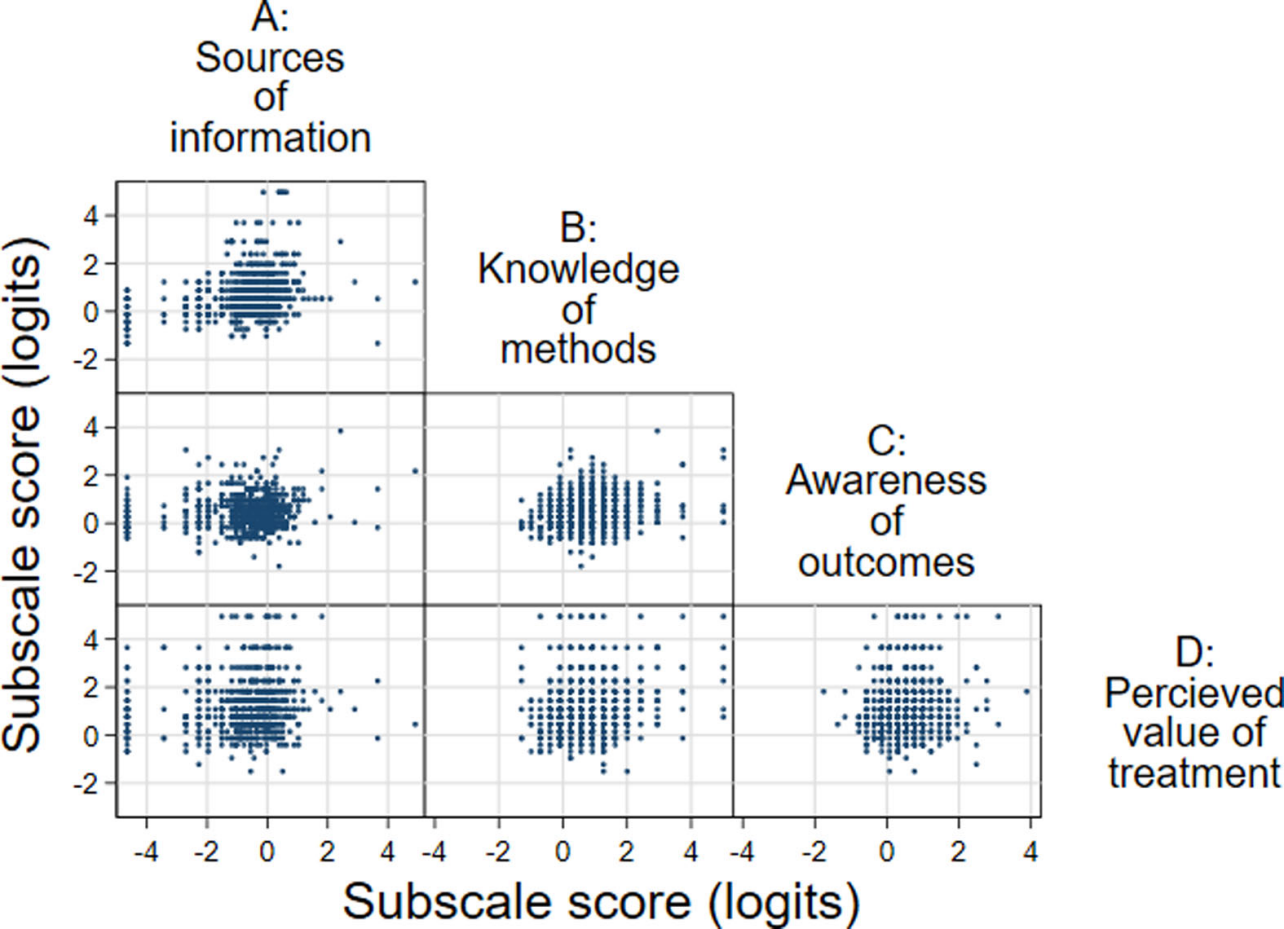
Scatterplot matrix of recalibrated subscale scores from the AGT-Eye instrument (*n* = 681 respondents).

Weak positive correlations were observed between each of the subscales (ρ = 0.08–0.31) (Fig. 2, Supplementary Table S7). People with higher scores on Subscale D (*Perceived value of treatment*) were slightly more likely to respond that they would take up gene therapy if it was available (ρ = 0.21; 95% confidence interval [CI], 0.14–0.28), and to agree with AGT-Eye items 8 and 9 in Subscale C (*Awareness of outcomes*), which are likely to coincide with positive beliefs about ocular gene therapy (see Supplementary Table S7).

## Discussion

Assessment of responses to the AGT-Eye from a large sample of Australians with IRDs revealed overall acceptable measurement properties across four subscales but also highlighted several shortcomings. Despite the large sample size, participants could not be adequately stratified according to level of knowledge of gene therapy or level of perceived value of treatment. Only a moderate degree of variation in responses could be explained by item and person measures on average, indicating that there are likely to be additional factors relating to attitudes and perceptions of gene therapy that must be explored. The most common participant response was to neither agree nor disagree with the statements in the AGT-Eye. This suggests that, on average, either the participants had insufficient knowledge on ocular gene therapy to form an opinion on the accuracy of these statements or the participants appreciated the complexity of the issues and therefore maintained equipoise. Along with the choice of items and wording, it is possible that the absence of strong beliefs about the gene therapy methods and outcomes contributed to the suboptimal stratification of participants into levels of the latent traits of interest.

### Comparison to Previous Studies

Several validated instruments are available to capture vision-related quality of life and mobility among people with IRDs.^27^^–^^29^ However, surveys specifically designed to capture attitudes toward genetic testing and gene therapy for IRDs have examined responses without investigating measurement properties.^30^^,^^31^ Decision making among people considering participating in an interventional gene therapy trials for X-linked retinoschisis and choroideremia has been investigated using rigorous qualitative methodology, as have the expectations, motivations, and barriers to participating in gene therapy trials among people with Leber's congenital amaurosis.^17^^,^^18^^,^^32^ To our knowledge, data captured during those interviews have not been used to generate a standardized questionnaire intended for repeated testing.

While developing scales intended to assess disease-specific quality of life among adults with IRDs, Prem Senthil et al.^33^ found that eight to 12 items were required to gain precise measurement of each domain of interest. Therefore, additional items in the *Knowledge of methods* and *Perceived value of treatment* AGT-Eye subscales may assist in the ability to distinguish between different levels of these traits. Domains identified in instruments intended to gauge attitudes toward non-ocular clinical trials and approved treatments include treatment expectations and positive beliefs, risk and negative expectations, safety, information needs, convenience, treatment efficacy and satisfaction, level of patient involvement, and perceptions of staff.^11^^,^^34^^–^^37^

### Strengths and Limitations

Measurement properties were assessed among a very large sample of respondents with a variety of IRDs and demographic characteristics, indicating that saturation of viewpoints has likely been reached. Measurement properties were assessed under the item response theory framework that assumes a reflective model (i.e., that causality flows from the latent trait of interest to the item responses).^38^ However, Subscale A (*Sources of information*) may be considered formative, meaning that causality flows from these items to the latent traits of attitudes and perceptions.^38^ Similarly, the items in Subscale B (*Knowledge of methods*) and Subscale C (*Awareness outcomes*) are also likely to impact attitudes and perceptions. Therefore, combining all items to generate a single score is unlikely to be appropriate.

Although the survey was reviewed for clarity among representatives of the target audience to investigate content validity during pilot testing,^20^ we cannot guarantee that all respondents understood the true nature of all questions. Administration of the AGT-Eye more than once per participant would have enabled assessment of test–retest reliability and measurement error and may have identified ambiguously worded items. This could be an avenue for future investigation. The items relating to knowledge of procedures and potential outcomes were written in relation to current treatment modalities, likely limiting time invariance. In addition, items relating to government funding, health insurance, and travel to other states for treatment may not be relevant in healthcare systems outside of Australia, making the instrument less generalizable to international populations. Items with less specific wording may have better captured the latent traits of interest. However, the inclusion of items that are specific to current treatment conditions is common in KAP surveys and may provide a method of assessing whether prospective recipients have fully understood the nature of ocular gene therapy procedures prior to making treatment decisions. These items will also highlight which topics may benefit from targeted educational campaigns.

### Future Research

The associations between the AGT-Eye and patient demographics and quality of life measures are currently under investigation. Based on the findings from the current analysis, scores from the recalibrated subscales and individual items will be utilized in that study. Each item will be scored from 1 to 5 (as described above) before generating subscale totals.^25^ Future iterations of the instrument would benefit from additional investigation into the constructs of interest at the development stage and rigorous piloting of a wider pool of items. Removal of the *Neither agree nor disagree* option may force respondents to make choices to improve the ability of the scales to stratify participants. Alternatively or additionally, an option of *I don't know* may also assist in separating people who have insufficient information from those who comprehend the issues but maintain equipoise in relation to potential outcomes.^25^ We recommend that future versions of the AGT-Eye be evaluated for test–retest reliability and responsiveness to changes in patient education. In addition to measuring the attitudes and perceptions of treatment, the collection of data on vision-related quality of life from people who do undergo gene therapy will be crucial to fully inform future prospective recipients of the potential benefits and harms.^10^^,^^33^

## Conclusions

The current version of the AGT-Eye may be useful as a KAP-like survey with four separate subscales. Responses to questions about treatment methods and potential outcomes from gene therapy for IRDs are likely to be revealing for clinicians tasked with providing informed consent and for clinical and public health workers tasked with advocacy and communication regarding ocular gene therapy. However, the utility of these items as part of a single instrument administered to measure change in the perceptions and attitudes of treatment decision makers over time may be limited.

## Supplementary Material

Supplement 1
