# Supplementary material for: Measurement Properties of the Attitudes to Gene Therapy for the Eye (AGT-Eye) Instrument for People With Inherited Retinal Diseases
Source: Transl Vis Sci Technol. 2022 Feb 8;11(2):14. doi: 10.1167/tvst.11.2.14 (PMC8842718; doi:10.1167/tvst.11.2.14)
Supplement: Supplement 1 [file tvst-11-2-14_s001.pdf]

Psychometric properties of the Attitudes to Gene Therapy for the Eye (AGT-Eye) instrument

Supplementary Table S1: Items in the Attitudes to Gene Therapy for the Eye (AGT-Eye) instrument

Subscale	Item	
A. Sources of information	2	I have obtained information about gene therapy from:<br>a. My ophthalmologist<br>b. Other medical or health professional<br>c. Registry (e.g., Australian Inherited Retinal Disease Register)<br>d. Research group<br>e. Newspapers<br>f. Internet<br>g. Social media<br>h. Patient support group<br>i. Family/friends
A. Knowledge of methods	1	I have good knowledge about gene therapy for inherited retinal diseases.
	3	I understand the difference between an experimental treatment provided in a clinical trial and a treatment that has already been approved by the Australian Government.
	4	Gene therapy for the eye is suitable at any stage of a person's life.
	5	Generally, gene therapy for inherited retinal disease is delivered to both eyes.
	6	Gene therapy for the eye is injected into the blood stream through the arm.
	7	Gene therapy and stem cell therapy are the same treatment.
D. Awareness of potential outcomes	8	Gene therapy for the eye can restore vision back to normal.
	9	Gene therapy for the eye is a treatment that may slow down the disease.
	10	Treatment complications to my eyes, such as permanent blindness, are possible with an approved gene therapy.
	11	Gene therapy in my eye may have side effects elsewhere in my body.
	12	Having gene therapy for their eye condition means a person will not pass on an eye condition to any children they may have in the future.
	13	I may not be eligible for financial or other government benefits if my gene therapy for my eye condition is successful.
	14	Gene therapy for inherited retinal diseases will require many years of follow-up with my eyecare practitioner.
	15	Receiving gene therapy for my inherited retinal disease means I won't be eligible for future genetic treatments.
	16	I will lose my privacy if I undergo gene therapy, and my data will be in the public domain.
	17	If I undergo gene therapy, it will affect my eligibility or terms of conditions in life, disability or health insurance in the future.
E. Value of treatment	18	The government should pay all costs of my gene therapy
	19	Government subsidy of my treatment would be an effective use of taxpayer money.
	20	If gene therapy for my condition was not available in my state I would consider travelling interstate to access it.
	21	My private health insurance should pay all out of pocket costs for my gene therapy.
	22	I would consider a payment plan for my gene therapy.

Supplementary Table S2: Classification of inherited retinal diseases

Widespread	Macular
ABCA4	Best vitelliform macular dystrophy
Fundus flavimaculatus	Bull's eye maculopathy
Abetalipoproteinaemia	Leber congenital amaurosis
Achromatopsia	Macular dystrophy
Rod monochromatism	Pattern dystrophy
Bietti Crystalline Dystrophy	Sorsby fundus dystrophy
Bull's eye retinal dystrophy	Stargardt disease
Central areolar choroidal dystrophy	X-Linked retinoschisis
CEP47	Malattia Leventinese
Choroideremia	
Cone-rod dystrophy	
X-linked cone rod dystrophy	
Cone dystrophy	
Congenital stationary night blindness	
Doyne honeycomb retinal dystrophy	
Familial exudative vitreoretinopathy	
Goldmann-Favre syndrome	
Gyrate atrophy	
PRPH2-associated retinopathy	
Retinal dystrophy	
Retinitis pigmentosa	
Laurence-Moon-Bardet-Biedl Syndrome	
Usher syndrome	
Vitreoretinochoroidopathy	

Supplementary Table S3: Demographics of included and excluded surveys amongst those with demographic data

	**Excluded** n=176	**Included** n=681	**Total** n=857	**p-value***
Age of respondent (years)				0.547
Range	16-84	18-93	16-93	
Mean (SD)	52.6 (16.5)	53.5 (15.8)	53.3 (16.0)	
Submission Type				0.204
Online	160 (90.9%)	593 (87.1%)	753 (87.9%)	
Paper	16 (9.1%)	80 (11.7%)	96 (11.2%)	
Phone interview	0 (0.0%)	8 (1.2%)	8 (0.9%)	
Respondent patient status, n (%)				<0.001
Adult patient	149 (84.7%)	639 (93.8%)	788 (91.9%)	
Parent, guardian, or caregiver	27 (15.3%)	42 (6.2%)	69 (8.1%)	
Gender, n (%)				0.140
Male	75 (42.6%)	327 (48.0%)	402 (46.9%)	
Female	96 (54.5%)	352 (51.7%)	448 (52.3%)	
Non-binary	0 (0.0%)	2 (0.3%)	2 (0.2%)	
I prefer not to say	1 (0.6%)	0 (0.0%)	1 (0.1%)	
Missing	4 (2.3%)	0 (0.0%)	4 (0.5%)	
Vision loss status, n (%)				0.083
Widespread	91 (51.7%)	525 (77.1%)	616 (71.9%)	
Macular	39 (22.2%)	156 (22.9%)	195 (22.8%)	
Missing	46 (26.1%)	0 (0.0%)	46 (5.4%)	
Highest level of education completed, n (%)				0.385
Primary school	7 (4.0%)	15 (2.2%)	22 (2.6%)	
Secondary school (Year 10 or above)	51 (29.0%)	223 (32.7%)	274 (32.0%)	
Trade certificate	34 (19.3%)	130 (19.1%)	164 (19.1%)	
Bachelor's degree	43 (24.4%)	172 (25.3%)	215 (25.1%)	
Post-graduate degree	32 (18.2%)	126 (18.5%)	158 (18.4%)	
I prefer not to say	8 (4.5%)	15 (2.2%)	23 (2.7%)	
Missing	1 (0.6%)	0 (0.0%)	1 (0.1%)	
Gross annual household income, n (%)				0.335
Less than $18 200	15 (8.5%)	42 (6.2%)	57 (6.7%)	
$18 201 - $37 000	22 (12.5%)	116 (17.0%)	138 (16.1%)	
$37 001 - $87 000	42 (23.9%)	168 (24.7%)	210 (24.5%)	
$87 001 - $180 000	44 (25.0%)	185 (27.2%)	229 (26.7%)	
More than $180 001	13 (7.4%)	61 (9.0%)	74 (8.6%)	
I prefer not to say	37 (21.0%)	109 (16.0%)	146 (17.0%)	
Missing	3 (1.7%)	0 (0.0%)	3 (0.4%)	
Would receive gene therapy if available now, n (%)				0.232
Very unlikely	1 (0.6%)	9 (1.3%)	10 (1.2%)	
Unlikely	2 (1.1%)	5 (0.7%)	7 (0.8%)	
Neutral	14 (8.0%)	43 (6.3%)	57 (6.7%)	
Likely	41 (23.3%)	122 (17.9%)	163 (19.0%)	
Very likely	112 (63.6%)	502 (73.7%)	614 (71.6%)	
Missing	6 (3.4%)	0 (0.0%)	6 (0.7%)	

* Two-sample t test (age) and Pearson's χ^2 test (categorical variables)

Supplementary Table S4: Frequency of responses to items in the Attitudes to Gene Therapy for the Eye (AGT-Eye) instrument (n=681).

Item	n (%) Strongly disagree	Disagree	Neither agree/disagree	Agree	Strongly agree
1	83 (12.2)	170 (25.0)	235 (34.5)	139 (20.4)	54 (7.9)
2a	158 (23.2)	143 (21.0)	122 (17.9)	175 (25.7)	83 (12.2)
2b	203 (29.8)	222 (32.6)	125 (18.4)	97 (14.2)	34 (5.0)
2c	182 (26.7)	176 (25.8)	136 (20.0)	141 (20.7)	46 (6.8)
2d	186 (27.3)	188 (27.6)	145 (21.3)	118 (17.3)	44 (6.5)
2e	225 (33.0)	205 (30.1)	142 (20.9)	98 (14.4)	11 (1.6)
2f	124 (18.2)	111 (16.3)	110 (16.2)	239 (35.1)	97 (14.2)
2g	249 (36.6)	196 (28.8)	122 (17.9)	84 (12.3)	30 (4.4)
2h	239 (35.1)	213 (31.3)	134 (19.7)	68 (10.0)	27 (4.0)
2i	211 (31.0)	167 (24.5)	154 (22.6)	121 (17.8)	28 (4.1)
3	8 (1.2)	22 (3.2)	59 (8.7)	274 (40.2)	318 (46.7)
4*	120 (17.6)	229 (33.6)	272 (39.9)	52 (7.6)	8 (1.2)
5	3 (0.4)	51 (7.5)	338 (49.6)	201 (29.5)	88 (12.9)
6*	21 (3.1)	68 (10.0)	421 (61.8)	102 (15.0)	69 (10.1)
7*	15 (2.2)	37 (5.4)	308 (45.2)	229 (33.6)	92 (13.5)
8*	18 (2.6)	58 (8.5)	348 (51.1)	202 (29.7)	55 (8.1)
9	1 (0.1)	6 (0.9)	202 (29.7)	381 (55.9)	91 (13.4)
10	10 (1.5)	67 (9.8)	388 (57.0)	188 (27.6)	28 (4.1)
11	15 (2.2)	88 (12.9)	416 (61.1)	148 (21.7)	14 (2.1)
12*	10 (1.5)	29 (4.3)	220 (32.3)	268 (39.4)	154 (22.6)
13	12 (1.8)	57 (8.4)	345 (50.7)	207 (30.4)	60 (8.8)
14	1 (0.1)	17 (2.5)	207 (30.4)	342 (50.2)	114 (16.7)
15	28 (4.1)	185 (27.2)	431 (63.3)	31 (4.6)	6 (0.9)
16*	17 (2.5)	33 (4.8)	152 (22.3)	302 (44.3)	177 (26.0)
17	44 (6.5)	182 (26.7)	368 (54.0)	73 (10.7)	14 (2.1)
18	14 (2.1)	100 (14.7)	275 (40.4)	187 (27.5)	105 (15.4)
19	3 (0.4)	14 (2.1)	126 (18.5)	307 (45.1)	231 (33.9)
20	14 (2.1)	44 (6.5)	102 (15.0)	273 (40.1)	248 (36.4)
21	18 (2.6)	65 (9.5)	284 (41.7)	188 (27.6)	126 (18.5)
22	13 (1.9)	48 (7.0)	196 (28.8)	309 (45.4)	115 (16.9)

* Reverse coded prior to tabulating
No shading = least frequent, darker shading = more frequent responses in categories of 15% width.

Supplementary Table S5: Eigenvalue of principal factors of the Attitudes to Gene Therapy for the Eye (AGT-Eye) instrument (n=681 participants, 30 items)

Factor	Eigenvalue		Proportion of variance	
	In this factor	Difference to next	In this factor	Cumulative
1	4.14	1.97	0.44	0.44
2	2.18	0.85	0.23	0.67
3	1.33	0.17	0.14	0.81
4	1.16	0.28	0.12	0.94
5	0.88	0.27	0.09	1.03
6	0.61	0.15	0.07	1.10
7	0.46	0.11	0.05	1.14
8	0.35	0.08	0.04	1.18
9	0.27	0.04	0.03	1.21
10	0.23	0.09	0.02	1.24
11	0.14	0.01	0.02	1.25
12	0.13	0.06	0.01	1.27
13	0.07	0.05	0.01	1.27
14	0.02	0.03	0.00	1.28

Analyzed using principal factor method for correlation between responses.
Responses coded 1-5 as recorded (no reverse coding or item response theory modelling prior to analysis).
Shaded cells indicate factors with eigenvalue >1.

Supplementary Table S6: Unrotated factor loadings for items of the Attitudes to Gene Therapy for the Eye (AGT-Eye) instrument (n=681)

Item	Factor*				Uniqueness
	1	2	3	4	
1	0.61	-0.17	0.07	-0.05	0.59
2a	0.53	-0.04	0.00	-0.14	0.69
2b	0.65	0.04	-0.18	-0.06	0.55
2c	0.62	0.05	-0.11	-0.13	0.59
2d	0.65	0.05	-0.21	0.03	0.54
2e	0.61	0.15	-0.15	0.05	0.58
2f	0.60	-0.27	-0.03	0.11	0.55
2g	0.59	0.09	-0.13	0.19	0.59
2h	0.64	0.04	-0.21	0.13	0.53
2i	0.55	0.07	-0.08	0.02	0.69
3	0.23	-0.31	0.20	-0.05	0.81
4†	0.27	0.17	0.25	-0.32	0.73
5	0.24	0.14	0.27	-0.27	0.78
6†	0.01	0.54	0.03	-0.24	0.65
7†	0.02	0.53	-0.05	-0.20	0.68
8†	0.11	0.49	0.12	-0.22	0.68
9	0.27	-0.23	0.32	-0.05	0.77
10	0.13	0.02	0.21	0.48	0.71
11	0.05	0.15	0.16	0.44	0.76
12†	-0.02	0.56	-0.04	-0.06	0.68
13	0.14	0.04	0.22	0.03	0.93
14	0.19	-0.08	0.21	0.16	0.89
15	-0.03	0.36	0.08	0.28	0.79
16†	0.10	0.51	0.04	0.18	0.69
17	0.03	0.33	0.19	0.35	0.73
18	0.08	0.26	0.33	0.11	0.81
19	0.16	0.02	0.46	-0.07	0.76
20	0.25	-0.25	0.36	-0.07	0.74
21	0.06	0.10	0.33	0.00	0.88
22	0.18	-0.19	0.29	-0.11	0.84

* Analyzed using principal factor method for correlation between responses. Four factors with an eigenvalue >1 explained 93.8% of total variance.
Responses coded 1-5 as recorded (no reverse coding for false statements† or item response theory modelling prior to analysis).
Unrotated loading of ≥0.3 shaded blue (positive loading) or yellow (negative loading). Items with low loading in all factors shaded gray.
Items with uniqueness ≤0.6 across these four factors shaded green.

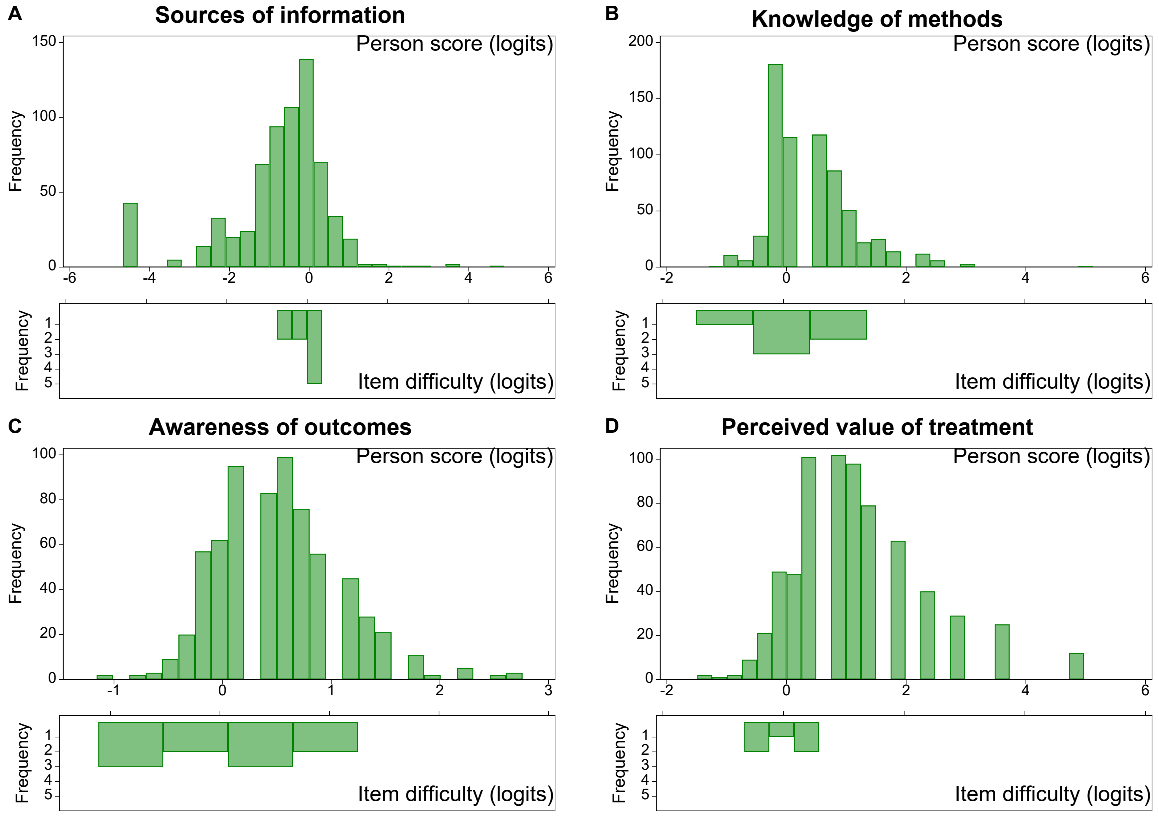

Supplementary Figure S1: Person-item maps for each original subscale of the Attitudes to Gene Therapy for the Eye (AGT-Eye) instrument (n=681 participants and 30 items). Rating scale models fitted for Subscales A and D; grouped rating scale models fitted with reverse-coded items grouped for Subscales B and C.

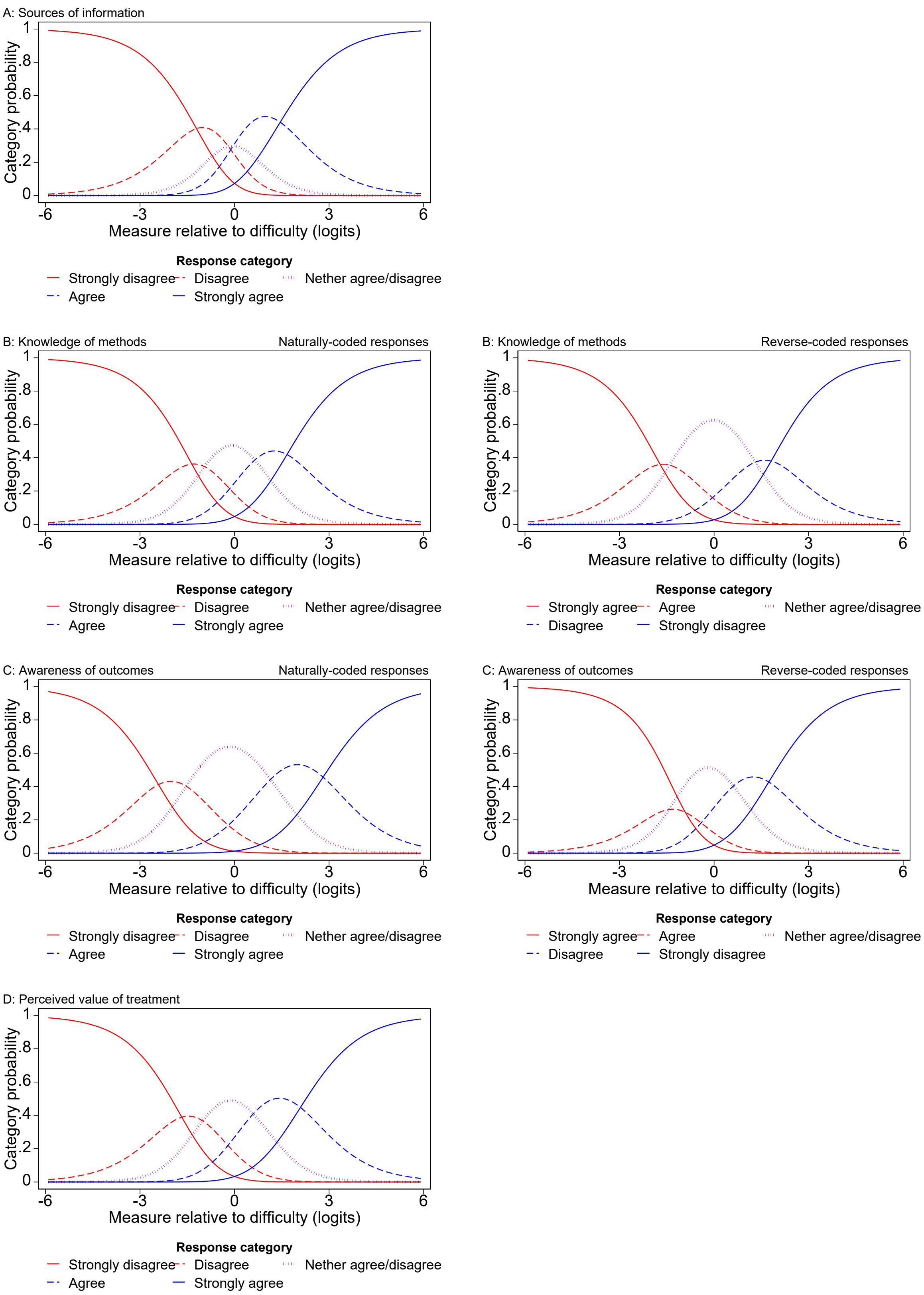

Supplementary Figure S2: Category response curves for original subscales of the Attitudes to Gene Therapy for the Eye (AGT-Eye) instrument.

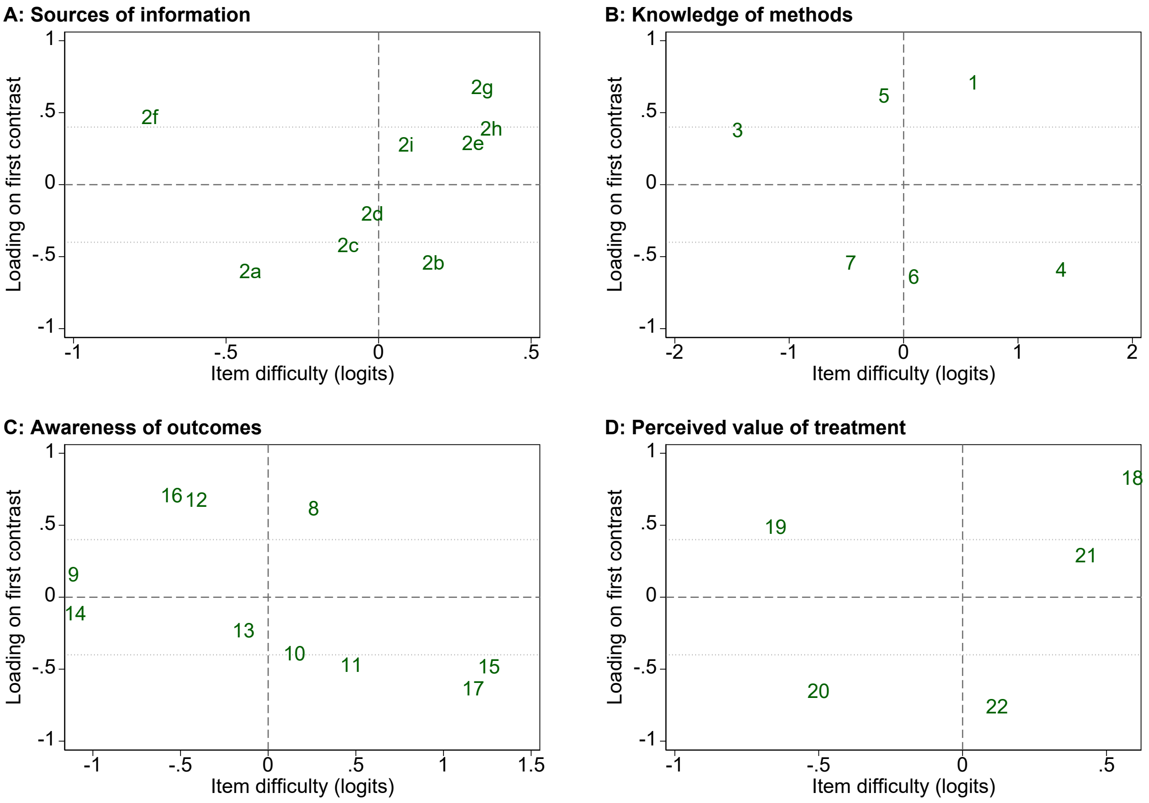

Supplementary Figure S3: Contrast loading plots for the first contrast from principal components analysis of model residuals from each original subscale of the Attitudes to Gene Therapy for the Eye (AGT-Eye) instrument. Rating scale models fitted for Subscales A and D; grouped rating scale models fitted with reverse-coded items grouped for Subscales B and C. Dotted horizontal lines at loading of -0.4 and 0.4. Numbers within plot area indicate item number.

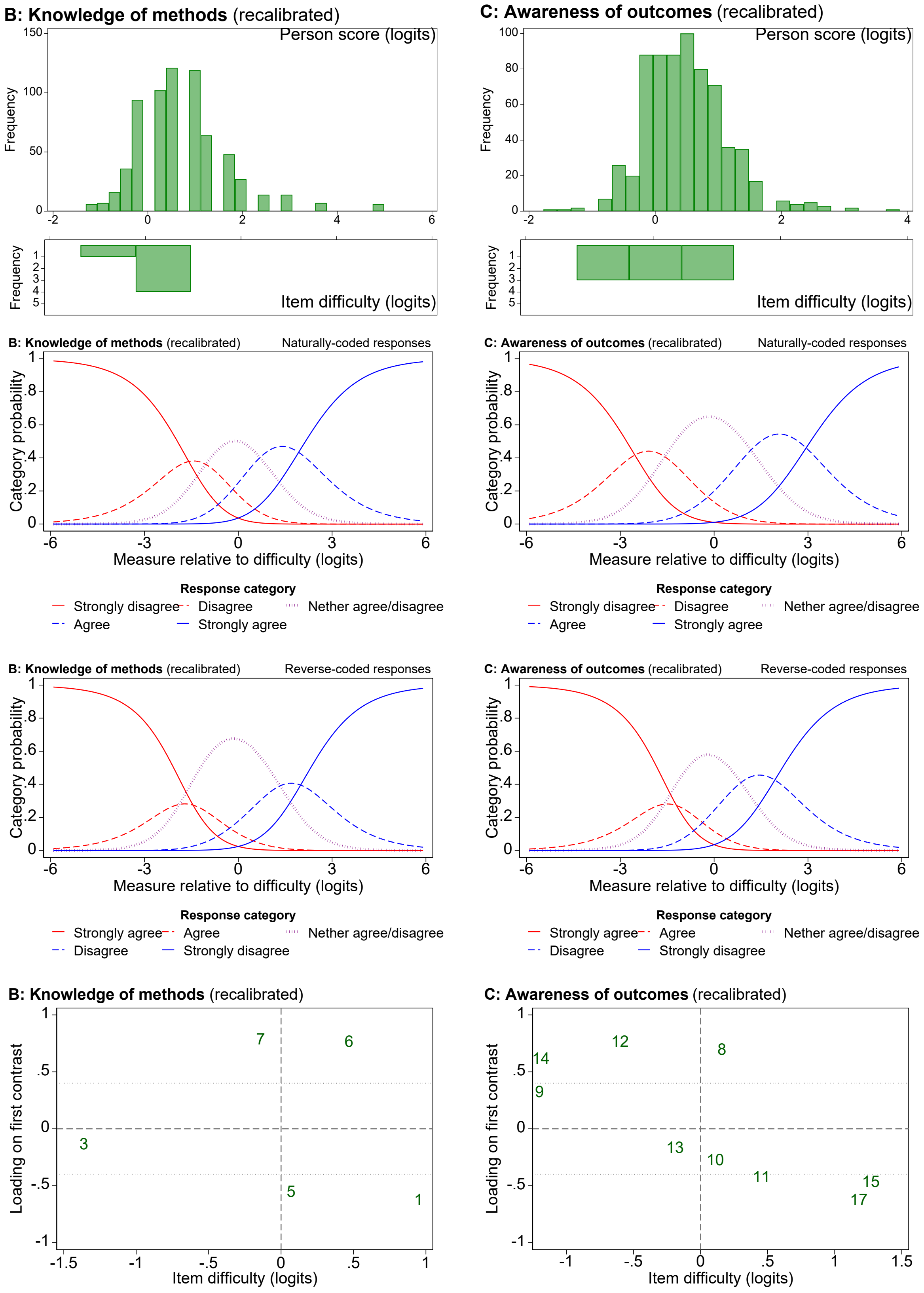

Supplementary Figure S4: Person-item maps, category response curves, and contrast loading plots for recalibrated subscales of the Attitudes to Gene Therapy for the Eye (AGT-Eye) instrument (n=681 participants and 28 items).

Supplementary Table S7: Correlation between recalibrated subscale scores (in logits) and each of the AGT-Eye items and demographic questions from the survey (scored as recorded).

Subscale	Item	Spearman's correlation coefficient (95% CI)			
		**A: Sources of information**	**B: Knowledge of methods**	**C: Awareness of outcomes**	**D: Perceived value of treatment**
**A** Sources of information	2a	0.58 (0.53, 0.63)	0.19 (0.12, 0.26)	0.06 (-0.01, 0.14)	0.12 (0.04, 0.19)
	2b	0.74 (0.70, 0.77)	0.19 (0.12, 0.26)	-0.01 (-0.09, 0.06)	0.05 (-0.03, 0.12)
	2c	0.68 (0.64, 0.72)	0.14 (0.06, 0.21)	-0.00 (-0.08, 0.07)	0.12 (0.04, 0.19)
	2d	0.72 (0.68, 0.75)	0.14 (0.06, 0.21)	0.04 (-0.04, 0.11)	0.05 (-0.02, 0.13)
	2e	0.70 (0.66, 0.74)	0.08 (0.00, 0.15)	-0.02 (-0.09, 0.06)	0.10 (0.02, 0.17)
	2f	0.60 (0.55, 0.65)	0.40 (0.33, 0.46)	0.18 (0.10, 0.25)	0.15 (0.07, 0.22)
	2g	0.68 (0.64, 0.72)	0.07 (-0.01, 0.14)	0.03 (-0.05, 0.10)	0.08 (0.00, 0.15)
	2h	0.74 (0.70, 0.77)	0.13 (0.06, 0.20)	0.02 (-0.05, 0.10)	0.03 (-0.05, 0.10)
	2i	0.64 (0.60, 0.68)	0.12 (0.04, 0.19)	0.00 (-0.07, 0.08)	0.08 (0.01, 0.15)
**B** Knowledge of methods	1	0.50 (0.44, 0.55)	0.68 (0.64, 0.72)	0.13 (0.06, 0.21)	0.13 (0.06, 0.21)
	3	0.11 (0.03, 0.18)	0.58 (0.52, 0.62)	0.18 (0.11, 0.26)	0.13 (0.05, 0.20)
	4*	0.18 (0.11, 0.25)	0.17 (0.10, 0.24)	0.02 (-0.06, 0.09)	0.14 (0.06, 0.21)
	5	0.13 (0.06, 0.20)	0.36 (0.30, 0.43)	-0.01 (-0.09, 0.06)	0.14 (0.06, 0.21)
	6*	-0.02 (-0.10, 0.05)	-0.45 (-0.50, -0.38)	-0.13 (-0.21, -0.06)	-0.06 (-0.13, 0.02)
	7*	-0.01 (-0.08, 0.07)	-0.56 (-0.61, -0.51)	-0.22 (-0.29, -0.15)	-0.06 (-0.14, 0.01)
**C** Awareness of outcomes	8*	0.05 (-0.03, 0.12)	-0.17 (-0.24, -0.10)	-0.38 (-0.44, -0.31)	0.10 (0.02, 0.17)
	9	0.17 (0.09, 0.24)	0.34 (0.27, 0.41)	0.42 (0.36, 0.48)	0.23 (0.16, 0.30)
	10	0.07 (0.00, 0.15)	0.11 (0.03, 0.18)	0.55 (0.50, 0.60)	0.08 (0.01, 0.15)
	11	0.01 (-0.07, 0.08)	-0.04 (-0.12, 0.03)	0.44 (0.38, 0.50)	0.04 (-0.04, 0.11)
	12*	-0.02 (-0.10, 0.05)	-0.32 (-0.39, -0.25)	-0.42 (-0.48, -0.36)	-0.06 (-0.14, 0.01)
	13	0.07 (-0.01, 0.14)	0.09 (0.02, 0.17)	0.41 (0.35, 0.47)	0.06 (-0.02, 0.13)
	14	0.12 (0.04, 0.19)	0.20 (0.13, 0.27)	0.53 (0.48, 0.58)	0.08 (0.01, 0.16)
	15	-0.10 (-0.17, -0.02)	-0.17 (-0.24, -0.10)	0.23 (0.15, 0.30)	0.01 (-0.06, 0.09)
	16*	0.07 (-0.01, 0.14)	-0.22 (-0.30, -0.15)	-0.01 (-0.09, 0.06)	-0.05 (-0.13, 0.02)
	17	0.00 (-0.07, 0.08)	-0.12 (-0.19, -0.05)	0.36 (0.29, 0.42)	0.05 (-0.03, 0.12)
**D** Perceived value of treatment	18	0.03 (-0.05, 0.10)	-0.04 (-0.11, 0.04)	0.00 (-0.07, 0.08)	0.53 (0.47, 0.58)
	19	0.10 (0.03, 0.17)	0.12 (0.04, 0.19)	0.07 (-0.00, 0.15)	0.68 (0.64, 0.72)
	20	0.15 (0.08, 0.22)	0.28 (0.21, 0.35)	0.15 (0.07, 0.22)	0.64 (0.59, 0.68)
	21	0.01 (-0.06, 0.09)	-0.02 (-0.09, 0.06)	0.07 (-0.00, 0.15)	0.60 (0.55, 0.64)
	22	0.10 (0.02, 0.17)	0.16 (0.09, 0.24)	0.13 (0.05, 0.20)	0.48 (0.42, 0.53)
Likely to take up gene therapy		0.12 (0.04, 0.19)	0.18 (0.11, 0.25)	0.04 (-0.03, 0.12)	0.04 (-0.03, 0.12)
Participated in research		0.07 (-0.01, 0.14)	0.11 (0.03, 0.18)	0.11 (0.04, 0.19)	0.11 (0.04, 0.19)
**Subscale scores** (in logits)					
A		1.00			
B		0.31 (0.24, 0.38)	1.00		
C		0.08 (0.00, 0.15)	0.23 (0.16, 0.30)	1.00	
D		0.14 (0.07, 0.21)	0.15 (0.08, 0.22)	0.13 (0.05, 0.20)	1.00

* No individual items were reverse coded prior to estimation of correlation coefficients for items; agreement with items 4, 6-8, 12 and 16 are likely to indicate poor knowledge.
Positive correlations shaded blue, negative correlations shaded yellow.
